# Molecularly and clinically related drugs and diseases are enriched in phenotypically similar drug-disease pairs

**DOI:** 10.1186/s13073-014-0052-z

**Published:** 2014-08-17

**Authors:** Ingo Vogt, Jeanette Prinz, Mónica Campillos

**Affiliations:** Institute of Bioinformatics and Systems Biology, Helmholtz Zentrum München, 85764 Neuherberg, Germany; German Center for Diabetes Research, 85764 Neuherberg, Germany

## Abstract

**Background:**

The incomplete understanding of disease causes and drug mechanisms of action often leads to ineffective drug therapies or side effects. Therefore, new approaches are needed to improve treatment decisions and to elucidate molecular mechanisms underlying pathologies and unwanted drug effects.

**Methods:**

We present here the first analysis of phenotypically related drug-disease pairs. The phenotypic similarity between 4,869 human diseases and 1,667 drugs was evaluated using an ontology-based semantic similarity approach to compare disease symptoms with drug side effects. We assessed and visualized the enrichment over random of clinical and molecular relationships among drug-disease pairs that share phenotypes using lift plots. To determine the associations between drug and disease classes enriched among phenotypically related pairs we employed a network-based approach combined with Fisher's exact test.

**Results:**

We observed that molecularly and clinically related (for example, indication or contraindication) drugs and diseases are likely to share phenotypes. An analysis of the relations between drug mechanisms of action (MoAs) and disease classes among highly similar pairs revealed known and suspected MoA-disease relationships. Interestingly, we found that contraindications associated with high phenotypic similarity often involve diseases that have been reported as side effects of the drug, probably due to common mechanisms. Based on this, we propose a list of 752 precautions or potential contraindications for 486 drugs.

**Conclusions:**

Phenotypic similarity between drugs and diseases facilitates the proposal of contraindications and the mechanistic understanding of diseases and drug side effects.

**Electronic supplementary material:**

The online version of this article (doi:10.1186/s13073-014-0052-z) contains supplementary material, which is available to authorized users.

## Background

Therapeutic drug intervention is widely used to treat diseases or their symptoms. However, drug therapy is often inefficient due to the poor understanding of the molecular causes of diseases or is associated with unwanted side effects. Therefore, new approaches aiming at improving drug treatment decisions and unveiling molecular mechanisms underlying diseases and drug actions are needed. In this regard, several computational methods that integrate experimentally and theoretically inferred molecular information of drugs and diseases, such as their associated gene expression profiles [1], drug targets, disease genes, and protein and compound structure [2], have been proposed. As a result, novel associations between drug and diseases, such as new indications and drug side effects [3], have been recognized. However, these approaches are limited to pre-existing and often incomplete molecular information and suffer from bias inherent to the experimental models [4].

As a consequence, alternative integrative approaches that rely on organismal phenotypes are emerging as valuable sources of information aiding the understanding of human pathologies. These methods avoid the aforementioned disadvantages of utilizing experimental molecular data as they deal with *in vivo* physiological information of the whole organism. For example, genome-wide association studies have identified multiple molecular determinants of diseases [5] and the analysis of disease symptoms from medical patient records has been shown to be able to capture disease comorbidities, predict disease progression and, most interestingly, molecular causes of diseases [6,7].

Furthermore, the observation that organismal phenotypes also carry information about molecular changes induced by system perturbations in mammals has been confirmed by numerous integrative analyses of phenotypic and molecular information. In particular, drugs sharing side effects tend to bind to common protein targets [8] and mouse models of functionally related genes often show similar phenotypes [9]. Likewise, genes associated with diseases that share symptoms are often functionally related [10,11]. In addition, comparative analyses of phenotypic information across species and perturbations have been successful in capturing novel disease-related molecular information. For example, the comparison of phenotypes between mouse models and human diseases has been shown to be an alternative to classical molecular integration methods for gene prioritization in diseases [12–14]. Moreover, an analysis of phenotype resemblance between drugs and mouse models has suggested that phenotype comparison between species could be used to predict novel drug-target interactions [15]. All these pieces of evidence demonstrate that approaches exploiting phenotypic information show considerable promise in assisting in the discovery of novel molecular mechanisms of diseases and drug action.

In this study we investigated if diseases and drugs related by similarity of symptoms and side effects are also mechanistically related and whether this phenotypic similarity can be exploited to improve our understanding of disease etiology, drug side effects, and current clinical indications and contraindications. We show that the comparative analysis of a comprehensive data set of phenotype information from drugs and diseases can yield insights into the molecular mechanisms involved in these perturbations and help to provide a rational guide for therapeutic drug treatment decisions. Based on our findings, we provide a list of 752 precautions or potential contraindications for 486 drugs.

## Methods

### Data resources

#### Thesauri and ontologies

Below we describe the construction of the thesauri we used to identify diseases, drugs, and phenotypic features within electronic documents. These thesauri group synonymous medical or chemical terms into concepts. For instance, in our phenotypic feature thesaurus the terms *calcinosis*, *calcinoses*, *tissue calcification*, *pathologic calcification*, and *calcification*, *pathologic* form the concept *calcinosis*. In addition, we describe how we obtained a phenotypic feature ontology that provides the required hierarchical relationships to calculate the semantic similarity between drugs and diseases based on their side effects and symptoms, respectively.

#### Disease thesaurus

We created a disease thesaurus for the recognition of human disease terms from text sources by collecting several existing medical thesauri integrated in the UMLS Metathesaurus (US National Library of Medicine, 2011). Our disease thesaurus includes concepts that are linked to MeSH (Medical Subject Headings, 2011), Online Mendelian Inheritance in Man (OMIM®, 2011), or ICD-9-CM (International Classification of Diseases, Ninth Revision, Clinical Modification, 2010) and are classified as pathologic function in the UMLS Metathesaurus. In addition, it contains all English synonyms provided by all vocabularies included in the Metathesaurus that do not require an additional license beyond the UMLS license. We provide a list of these vocabularies as supporting information (Additional file 1).

#### Drug thesaurus

In order to recognize drug names from electronic documents, we created a drug thesaurus by integrating different sources of drug names. It consists of a collection of records each of which contains all synonyms for a given drug. Our drug thesaurus is mainly based on the chemical aliases from STITCH 2 [16], which provides an extensive source of names for drugs and other chemicals and their synonyms mapped to PubChem compound identifiers (CIDs). Each CID in STITCH thus represents a distinct record grouping the different names of a chemical. We complemented the STITCH 2 chemical name data with additional drug names and their synonyms from PubChem [17], Drugbank [18], KEGG DRUG [19], Anatomical Therapeutic Chemical (ATC) classification system, and Unique Ingredient Identifiers (UNII; from the FDA Substance Registration System [20]) (versions 2011). To map the additional sources to the STITCH data we preferentially used PubChem CIDs. If these identifiers were not available, we used other existing chemical identifiers from ChEBI (Chemical Entities of Biological Interest) and KEGG ID. If none of these identifiers matched to STITCH synonyms, we tried to match first the preferred name of the additional name source to our thesaurus. If this was not successful, we tried to match one of its synonyms (see Table S1 in Additional file 2 for details). For each record from the additional name sources that could not be matched in this way, we created a new record in our chemical name thesaurus. After this, we selected and kept only those records corresponding to therapeutically active molecules. To that aim, we collected all active ingredients listed by the US Food and Drug Administration (FDA), the European Medicines Agency (EMA), ATC classification system, RXNORM, and the electronic Medicines Compendium (eMC [21]). By matching the list of 6,653 active ingredient names to our chemical names thesaurus we obtained 3,943 records that correspond to therapeutically active molecules. These records constitute our final drug thesaurus, with 832 of them representing newly created drug records not contained in STITCH. In total, this thesaurus contains 157,930 distinct names of drugs. As reported in Table S1 in Additional file 2, 77.65% of these names are provided by STITCH; 1.89% more were mapped via chemical identifiers from PubChem, ChEBI, and KEGG. The remaining drug names were assigned based on preferred names (17.50%), other synonyms (2.86%) or manually (0.1%). By mapping the drug thesaurus to drug labels we extracted the therapeutic agents described in these labels. We then selected those drug labels with only one therapeutic agent. Throughout this manuscript we use the term 'drug' to refer to such a therapeutic agent and consider drug products that contain multiple active ingredients as drug combinations.

As stated in the Methods subsection 'Extraction of phenotypic data for diseases and drugs', we observed that drug names were correctly identified in all drug-related electronic documents for a random sample of 10 drugs covering all sources of drug side effect data.

#### Phenotypic feature thesaurus

Furthermore, we compiled a specific thesaurus for the extraction of both disease symptoms and drug side effects from electronic documents. We collected all UMLS concepts that are linked to the Medical Dictionary for Regulatory Activities (MedDRA; version 13.0, 2010) terminology and classified as either '*Anatomical Abnormality*', '*Finding*', or '*Natural Phenomenon or Process*'. Then, we gathered all terms that were also associated with these concepts from MedDRA and all vocabularies included in the Metathesaurus that do not require an additional license beyond the UMLS license. We provide a list of these sources in Additional file 3. Finally, terms were subjected to a set of filters and modification rules to optimize concept annotation [8,22–24]. This included suppression rules such as removing ambiguous terms or terms with more than 10 words. We removed terms based on term types (given by the UMLS Metathesaurus) suggested to be removed for concept recognition purposes in text [22]. These term types identify, for example, synonyms inappropriate for recognizing the original concept ('*Abdomen*' as synonym of '*Malignant neoplasm of abdomen*'), potential ambiguity of abbreviations ('*KD*', '*KS*'), or terms that contain meta-data unsuitable for text processing ('*Agoraphobia [Disease/Finding]*') (see section 7.1 in [22]). This step excluded 32,837 terms. Furthermore, to increase computational speed we discarded terms with many words, such as '*tuberculosis of intestines and mesenteric glands*, *bacteriological or histological examination unknown at present*' as it is unlikely to find an exact match for these terms to free natural language. Overall, we discarded 11,158 terms that contained more than 10 words. Then, we applied rewrite rules to account for variability of spelling and phrasing by conversion to lower case, variation in the possessive case ('*crohn's disease*' - '*crohn disease*' *-* '*crohns disease*'), and syntactic uninversion ('*pharyngitis*, *infective*' - '*infective pharyngitis*'). Both possessive case variations and syntactic uninversion have already been found to be effective and to not produce terms not corresponding to the original terms [24]. Such rewritten terms were added as synonyms in addition to the original terms. We added a syntactic uninversion of a term if that term contained one comma followed by a space and did not contain a preposition or conjunction [24]. In total, 40,228 terms were added by this step. By adding possessive case variations we further extended the thesaurus by 13,250 terms. All suppression and rewrite rules have been implemented as scripts for automation. In total, our phenotypic feature thesaurus contains 33,038 concepts covering 101,672 individual English terms.

#### Phenotypic feature ontology

The calculation of semantic similarity requires an ontology that provides hierarchical relationships between terms. For this purpose, we modified MedDRA's hierarchical organization that groups terms into four levels of specificity: *System Organ Classes* (SOCs, 26), *High-Level Group Terms* (HLGTs), *High-Level Terms* (HLTs), and *Preferred Terms* (PTs). SOCs represent the most general and PTs the most specific level. Originally, there is also a fifth level called *Low-Level Terms* (LLTs) that contains synonymous terms of the PT level including the PTs themselves. Because there is no clear hierarchical relationship between the PT and the LLT level, we merged the LLT level with the PT level. Furthermore, the MedDRA ontology links terms vertically and horizontally, so that terms can be linked across multiple SOCs. Only the SOC *Investigation* is kept separate and thus represents an isolated subtree in the ontology only connected to the root. This prevents a meaningful association between, for example, signs as '*blood glucose level increased*', a successor of '*Investigations*', with related terms such as '*hyperglycemia*'. Interestingly, MedDRA incorporates Standardized MedDRA Queries (SMQs), which represent groups of terms across the entire ontology including the SOC '*Investigations*' related to a defined medical condition (such as '*Hyperglycaemia/new onset diabetes mellitus*'). To overcome the issue of the isolated '*Investigations*' subtree, we thus selected SMQs linking terms belonging to the '*Investigations*' subtree to terms of other SOCs as additional relationships into our modified version of the MedDRA ontology. This yielded 61 SMQs from which we manually removed the SMQs '*Lack of efficacy/effect*' and '*Haemorrhage terms (excl laboratory terms)*', which we deemed irrelevant in the given context. We provide a list of the selected SMQs in Additional file 4. For the purpose of calculating phenotypic similarity between drugs and diseases, each concept from our phenotypic feature thesaurus is identified by its most specific MedDRA term. This is possible because we selected only those concepts from the UMLS Metathesaurus that contained at least one MedDRA term.

#### Extraction of phenotypic data for diseases and drugs

All phenotypic data were obtained from disease- and drug-related electronic documents provided by publicly accessible sources (in 2012) using a two-step semi-automatic text mining approach. We matched each term in our thesauri to the appropriate text sections making use of regular expressions. In addition, as explained in more detail later in this section, we devised a post-processing step based on manual inspection of false positives in order to reduce the number of false positive annotations resulting from the initial matching step. For diseases we had two different kinds of documents. OMIM provides a single text file (omim.txt.Z) that contains structured information on genes and disease phenotypes from which we extracted the entry titles for disease identification and the clinical synopsis sections listing sign and symptom data of diseases. The web resources '*The Merck Manual of Diagnosis and Therapy*' and '*The Merck Manual Home Health Handbook*' [25,26], 'A.D.A.M. Medical Encyclopedia' via MedlinePlus [27], and '*CureResearch*' [28] list available disease descriptions via tables of contents. The entries in these tables of content were used for disease identification while we extracted from each disease description the section describing signs and symptoms. Afterwards, the disease thesaurus was employed to identify the disease names and the phenotypic feature thesaurus was used to recognize signs and symptoms listed in the corresponding sections. For this automated annotation step we carefully designed regular expressions based on repeated manual analysis of the annotation results.

Drug-side effect information was extracted from public electronic document directed at health care professionals or the general public such as drug labels (FDA), drug monographs (BC Cancer Agency, Canada), summaries of product characteristics and assessment reports (eMC, UK, and EMA), and clinical report data from the MedEffect database, a health product safety database from Canada. While the latter contains drug-side effect data directly coded with MedDRA, the other drug documents contain specific sections devoted to reporting side effect information. We extracted these specific sections and the listed active ingredients and used our phenotypic feature thesaurus to identify side effects and the drug thesaurus to identify drug names from the active ingredient list. Specifically, we only collected side effect data from labels containing only one active ingredient, as we were only interested in the side effects caused by a single drug alone. The automated annotation step for drugs was conducted in the same way as for diseases. To further enhance the annotation results we devised a post-processing step that uses a different set of regular expressions aimed at identifying problematic or false positive concept annotations. For example, recognized concepts can actually be negated such as in '*absence of < concept >*', '*will/do/does not cause/develop < concept >*', '*without < concept >*', and '*never develop(s) < concept >*'. As our semantic similarity measure is not designed to take the absence of a given feature into account, we discarded all negated concepts for the presented analysis. Similarly, we discarded concept annotations that would lead to false drug-side effect or disease-symptom associations such as '*falsely attributed to < concept >*', '*for/in people/patients with < concept >*', '*mistaken for < concept >*'.

In order to assess our annotation performance, we manually evaluated the annotation results for 10 randomly selected drugs and diseases that covered all sources of phenotypic information. For a given drug, the evaluation of precision was conducted in the following fashion. First, we assessed each concept match individually. Then, concept annotations were pooled from all labels of the given drug and considered true if they were true at least once. Recall was estimated by manually extracting phenotypic information from all labels and subsequent comparison to automatically matched concepts. This evaluation scheme was also applied to disease annotations. We found that, on average, precision and recall was 96% and 90.3% for drugs and 98% and 65.7% for diseases. Within the randomly selected disease annotation data we also studied the prevalence of annotations that were less specific than what was stated in the document. Examples of these cases are '*abdominal cramps*' versus '*lower abdominal cramps*', '*corneal dystrophy*' versus '*central corneal dystrophy*', or '*polydactyly*' versus '*preaxial polydactyly*'. This can be due to the fact that the more specific term is not present in our phenotypic feature thesaurus or to misspelled terms. We found that about 8.3% of all obtained disease annotations represented less specific concepts. As the degree of specificity loss depends on each case individually, it is not possible to estimate the overall impact of this issue. In general, annotation of less specific phenotypic features might decrease the observed overall similarity between phenotypes. Finally, we also utilized the randomly selected examples to assess whether the assignments of the electronic documents to diseases and drugs in our thesauri were correct. We found that precision was 100% in both drugs and diseases. For drugs, we correctly identified only one active ingredient in each of the considered drug labels.

#### Protein-protein interaction network

For the analysis of shortest distances between disease gene products and drug targets we employed a protein-protein interaction (PPI) network of functionally interacting proteins based on STRING 9 PPI data [29]. Here, a functional interaction refers to pairs of proteins that interact physically or belong to the same biological pathway. To select these interactions, we obtained interaction data from experimental or database sources with a minimum confidence score of 0.7, the suggested score threshold to identify high confidence protein interactions [30,31]. In total, our PPI network consisted of 8,269 nodes and 256,012 interactions.

#### Disease genes

Information on disease genes was taken from DisGeNET [32,33]. We only considered data from OMIM. Overall, we collected 2,667 disease-gene associations between 1,794 genes and 1,815 diseases. Of these, 642 diseases have associated genes whose products are part of our PPI network.

#### Drug targets

We extracted drug targets from the STITCH 3 database that have a confidence score higher than 0.7 [34]. After the exclusion of indirect associations, 10,060 drug-target pairs remained. These pairs consist of 1,654 unique targets for 1,636 drugs (98.14%); 939 drugs have targets that are included in our PPI network (Table 1).

**Table 1:** **Overview of different sets of diseases, drugs, and drug-disease pairs**

	**Diseases**	**Drugs**	**Drug-disease pairs**
Disease/drug-feature pairs	55,031	155,973	-
With phenotypic features	4,869	1,667	8,116,623
With molecular information linked to human PPI	642	939	602,838
Drug-ADR-disease pairs/with PPI	1,294/76	736/619	34,467/1,442
Indications/with PPI	532/57	786/212	2,637/311
Contraindications/with PPI	229/23	948/58	2,230/61
Clinical trials/with PPI	813/68	1,022/197	7,744/381
In phenotypic similarity network	2,565	957	7,368
In network, with MeSH disease level 1	1,648	-	-
In network, with ATC information	-	805	-
In network, with MeSH disease level 2/drug MoA	939	379	1,352

ADR, adverse drug reaction MoA, mechanisms of action.

#### Known associations: adverse drug reaction-disease pairs

We selected those disorders reported at least once as a drug side effect in our drug-side effect data. Then we collected all drugs that have at least one of these selected diseases annotated as a side effect. This yielded a set of 34,467 drug-side effect relationships composed of 736 diseases and 1,294 drugs. We termed these relationships drug-adverse drug reaction (ADR)-disease pairs or ADR-disease relationships.

#### Known associations: indications and contraindications

The National Drug File - Reference Terminology (NDF-RT) is an extended version of the VHA National Drug File (NDF) and contains information on drugs approved in the US. We obtained the public version of the NDF-RT (accessed 2 May 2012) and extracted information on indications (attributes *may_prevent*, *may_treat*, and *induces*) and contraindications (attribute *CI_with*) for drugs and diseases included in our drug and disease thesaurus, respectively. In total, we collected 2,230 drug-disease contraindications and 2,637 indications.

#### Known associations: clinical trials

We obtained the 2011 version of the database for aggregate analysis of ClincalTrials.gov (AACT) [35] provided by the Clinical Trials Transformation Initiative (CTTI). From this database we extracted 7,744 drug-disease relationships from phase 3 and 4 trials for a single drug with a primary purpose of basic science, prevention, supportive care, or treatment.

### Phenotypic similarity measure

We assess phenotypic similarity by means of a semantic similarity measure based on the approach introduced by Resnik, which has been shown to perform well in several similar biomedical scenarios [36,37]. This approach assigns an information content (IC) to each ontology term as a measure of specificity. Then, the similarity between two terms is determined by the maximum IC of all common ancestor terms. This common ancestor is termed the most informative common ancestor (MICA). Commonly, the IC is derived from the annotation frequencies of ontology terms within a given corpus [37]. To avoid any annotation bias we employed an alternative approach that considers terms with less hyponyms (children in the hierarchy) as more specific [38]. Thus, all PTs in the MedDRA ontology obtain the maximum information content of 1, while the IC gradually decreases while traversing the hierarchy towards the root node, depending on the number of hyponyms. The root node subsumes all ontology terms and therefore is assigned the minimum IC of 0. The formula for IC calculation is as follows [38]:

1 $$ \mathrm{I}{\mathrm{C}}_{term}=1-\frac{ \log \left( hypo(term)+1\right)}{ \log (N)} $$

where *term* is a given ontology term, *hypo(term)* gives the number of all of its hyponyms, and *N* is the total number of terms in the ontology. In order to decrease the influence of frequent and redundant terms, we introduced weights accounting for annotation frequency and co-occurrence in both drugs and diseases, respectively. For example, the frequency weight of a disease symptom is defined as the negative natural logarithm of the fraction of diseases the term is annotated to.

For the co-occurrence weight we used the negative natural logarithm of the Jaccard index:

2 $$ J\left( A, B\right)=\frac{\left| A\cap B\right|}{\left| A\cup B\right|} $$

where *A* and *B* represent the sets of drugs or diseases a term is annotated to. For a given side effect or symptom the co-occurrence weight is then calculated as its average negative natural logarithm of the Jaccard index across all side effects and symptoms, respectively. This avoids giving too much weight to terms that commonly occur together. The similarity score between a side effect and a symptom is then calculated as the product of the IC of the MICA and the minimum of the overall weights in order to emphasize phenotypic effects specific in both diseases and drugs:

3 $$ {s}_{ad{ r}_i, sympto{m}_j}= I{C}_{MIC{ A}_{ad{ r}_i, sympto{m}_j}}\ast min\left({f}_{ad{ r}_i}\ast {c}_{ad{ r}_i},{f}_{s ympto{m}_j}\ast {c}_{s ympto{m}_j}\right) $$

where *f*_*adr/symptom*_ and *c*_*adr/symptom*_ refer to the aforementioned frequency and co-occurrence weights of the annotated side effect and symptom that are compared. The final overall phenotypic similarity between a drug and a disease is calculated as follows. For each side effect the best matching symptom is determined as identified by the highest similarity score:

4 $$ bes{t}_{ad{ r}_i}= max\left({s}_{ad{ r}_i, sympto{m}_1},{s}_{ad{ r}_i, sympto{m}_2},\dots, {s}_{ad{ r}_i, sympto{m}_m}\right) $$

Analogously, for each disease symptom the side effect yielding the highest similarity score among all side effects of the drug is considered as best match:

5 $$ bes{t}_{s ympto{m}_j}= max\left({s}_{ad{ r}_{1,} sympto{m}_j},{s}_{ad{ r}_{2,} sympto{m}_j},\dots, {s}_{ad{ r}_n, sympto{m}_j}\right) $$

The final phenotypic similarity is derived by summing the individual similarity scores from all best matches (Equations 4 and 5) and normalizing by the number of side effects and symptoms:

6 $$ similarity\left( drug, disease\right)=\frac{{\displaystyle \sum bes{t}_{ad{ r}_i}}+{\displaystyle \sum bes{t}_{sympto{ m}_j}}}{n+ m} $$

where *n* and *m* denote the number of side effects and symptoms, respectively.

### Phenotypic network analysis

#### Network generation

We generated a network of phenotypically similar diseases and drugs consisting of 2,565 diseases and 957 drugs linked by 7,368 edges. We employed the Pareto functionality provided by KNIME [39] to obtain a phenotypic similarity score threshold that optimizes the enrichment and precision for drug-disease pairs with a shortest distance of 0 and 1. For this optimization, we made use of pairs containing drugs and disease with molecular links (602,838 drug-disease pairs). These pairs include clinical drug-disease associations of all types, that is, indications, clinical trials, contraindications and ADR-disease, however, not all drug-disease associations are linked to molecular information. The final score threshold was 2.004.

#### Identification of communities

For the entire phenotypic drug-disease network a community detection was carried out with igraph employing the multi-level modularity optimization algorithm [40,41]. For a given partitioning of a network into distinct communities the modularity quantifies the extent to which there are more or less edges falling within the given communities compared to an equivalent network with edges placed at random [42]. The applied algorithm creates communities without any pre-defined restrictions on number or size of possible communities. It does so by iteratively grouping nodes into communities only if this increases the overall modularity. We utilize the term community instead of cluster to distinguish it from a different network property [43].

#### Disease and drug class enrichment in network communities

We chose the MeSH disease classification system to classify the diseases in our dataset. We could map 1,648 out of 2,565 (64.25%) diseases (Table 1) to the 2011 MeSH disease classification system by using information provided in the UMLS Metathesaurus. The first and thus most general MeSH disease classification level was used to determine class enrichment in communities using Fisher's exact test (Benjamini-Hochberg correction, false discovery rate (FDR ≤0.01)). Overall, 26 disease classes were present in the mapping results and subsequently considered for the enrichment analysis (diseases of the MeSH categories C01 to 20, C22 to 26, and F03). Analogously, we used ATC Anatomical Main Group classifications to classify drugs. We utilized the ATC codes-drug associations provided by STITCH, KEGG DRUG, and additionally we mapped active ingredient names obtained from the ATC Index [44] to our drug thesaurus. Out of 957 drugs, 805 (84.1%) could be annotated with one or more of 13 ATC classes (ATC group '*Various*' was not considered in this analysis). After mapping, enrichment of ATC classes among the identified network communities was determined using Fisher's exact test with Benjamini-Hochberg correction (FDR ≤0.01) for multiple testing.

#### Enrichment of disease class-drug class combinations

To allow a more specific analysis of disease classes we grouped diseases using the second more specific level of the MeSH disease classifications system. Drugs were assigned MeSH classes based on the level below '*Molecular Mechanisms of Pharmacological Action*' (D27.505.519). The MeSH mapping for drugs was obtained via mapping first our drug thesaurus to names of chemicals in the UMLS Metathesaurus and subsequently retrieving UMLS 'is a' relationships linking the recognized drugs to MesH. Then, over-representation of disease- and drug-class combinations within the phenotypic drug-disease network was assessed compared to the overall set of drug-disease pairs. Here, over-representation was established by using Fisher's exact tests followed by Benjamini-Hochberg correction and applying a threshold of FDR ≤0.05.

## Results

### Phenotypic similarity of disease-drug pairs

To analyze the phenotypic relations between drugs and diseases we collected phenotypic information for 1,667 drugs and 4,869 human diseases by annotating side effects and signs and symptoms with a medical ontology based on the MedDRA (see Methods). In total, we extracted 155,973 drug-side effect and 55,031 disease-symptom pairs (Table 1).

Next, we assessed the phenotypic similarity between all 8,116,623 pairwise drug-disease combinations. We used an approach that evaluates the average semantic similarity between all best matching pairs of side effects and symptoms, thus avoiding the bias towards drugs and diseases with many phenotypic features on the highest ranking drug-disease pairs (Figure S2 in Additional file 2). The semantic similarity measurement has been adapted from the method introduced by Resnik [45] combined with a weighting scheme to downweight frequent and correlated terms [8] (see Methods for details).

### Enrichment of short functional distances

**Figure 1:**
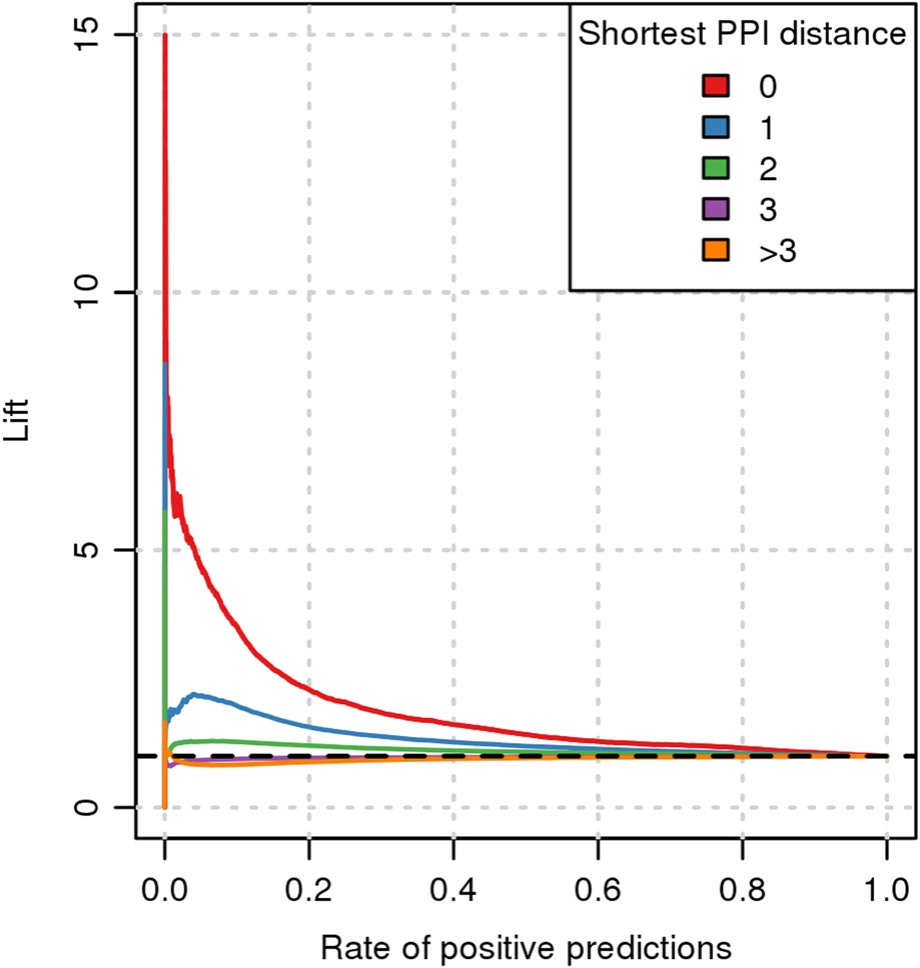
**Drug-disease pairs related by molecular mechanism exhibit similar phenotypes.** Enrichment of functional distances between drug targets and disease proteins of drug-disease pairs with similar phenotypes. 'Rate of positive prediction' refers to the list of drug-disease pairs sorted by decreasing phenotypic similarity. For each measurement all pairs with equal or higher similarity scores were taken into account. The dashed black line indicates the performance expected at random. The lift for any given interval starting at a rate of positive predictions of 0 is calculated by dividing the precision by the quotient of the total number of positives and negatives. If the order of the list of drug-disease pairs were random, the precision would always be equal to the quotient of the number of positives and negatives. Thus, the lift for random expectation is always 1.

Based on recent findings confirming that phenotypic information of drugs and diseases conveys molecular information [8,10], we first explored whether similarity of drug and disease phenotypes can be explained by common or related molecular mechanisms. We collected all pairs formed by the combination of 939 drugs and 642 diseases (Table 1) for which associated proteins in a human PPI network were available. Then, we calculated the shortest distance in this PPI network between the known drug targets and disease-associated proteins (see Methods). Next, we defined five distance categories, namely 0, 1, 2, 3, and >3, and measured the enrichment of each distance category over random expectation (lift; Figure 1) for increasing values of phenotypic similarity. As shown in Figure 1, drug-disease pairs associated with the same protein (distance 0) or with interacting proteins (distance 1) are strongly enriched among pairs with high phenotypic similarity. Notably, the enrichment diminishes with increasing distance between drug targets and disease-related gene products. Thus, we conclude that molecularly related diseases and drugs tend to share phenotypes and that the functional distance of affected proteins in the human PPI network influences the observed phenotypic similarity.

### Phenotypic disease-drug network

In order to analyze drugs and diseases with high phenotypic similarity in more detail, we constructed a phenotypic disease-drug network containing the pairs with high semantic similarity. For that, we employed a similarity score threshold that optimized the enrichment and precision for drug-disease pairs with common or related molecular mechanisms (distance 0 or 1) by means of Pareto optimization [46]. For this optimization, we only considered drugs and diseases with molecular information that could be linked to the PPI network, yielding 602,838 drug-disease pairs in total (Table 1). This network consists of 7,368 edges linking 2,565 diseases to 957 drugs, covers 52.7% and 57.4% of all diseases and drugs in our set, respectively, and contains the top 0.1% disease-drug pairs with highest phenotypic similarity.

### Enrichment of disease and drug classes in network communities

**Figure 2:**
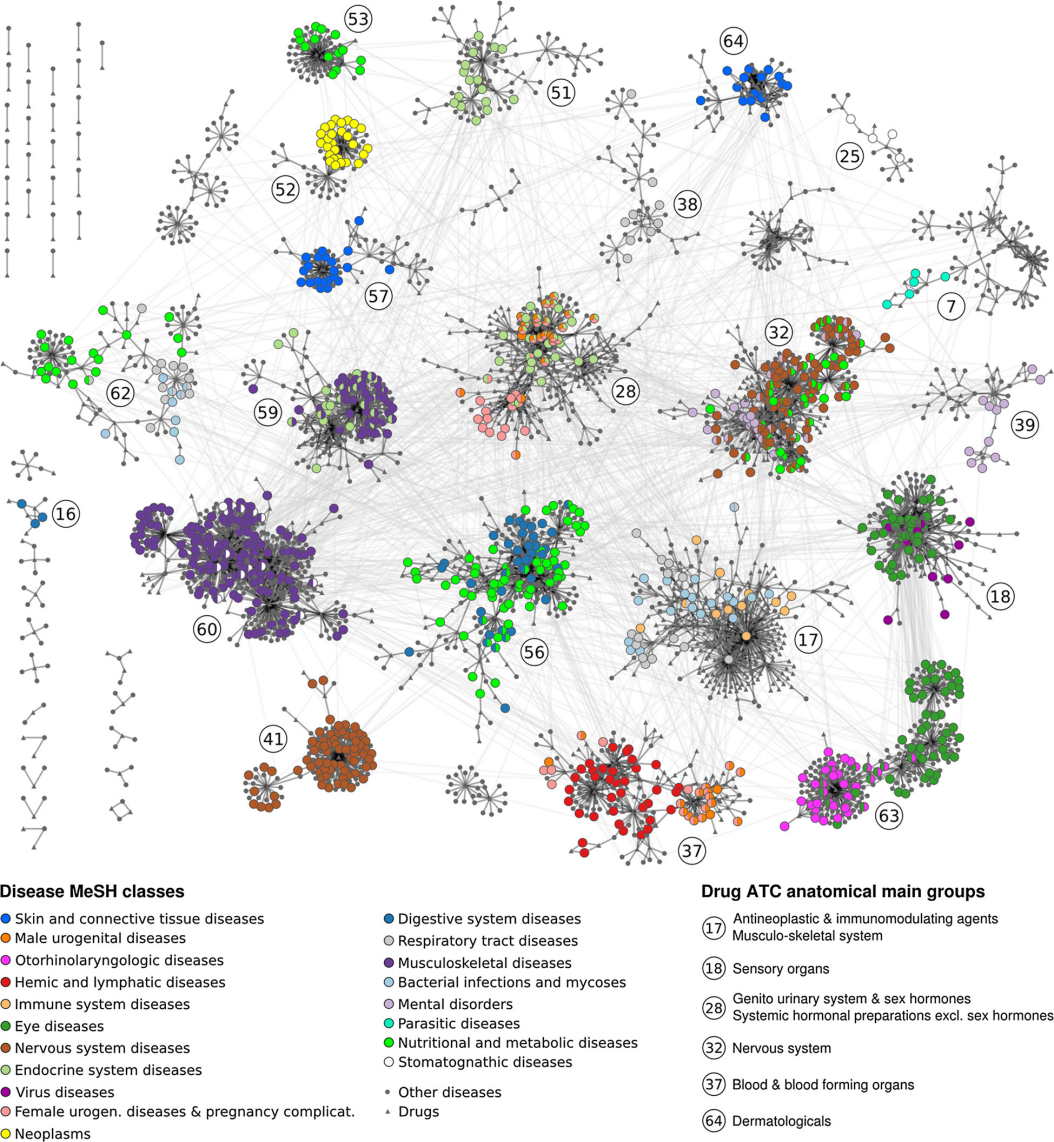
**Enrichment of disease and drug indication classes within communities of the phenotypic similarity network.** The modular organization of the phenotypic similarity network is shown. The communities with significantly interconnected diseases (circles) and drugs (triangles) are highlighted. The communities with enriched disease or drug classes are numbered. The enriched disease classes within the communities are marked by increased size and different colors, while the enrichment of drug classes is reported in the lower right corner.

In a first step, we sought to acquire a global view of the connections between disease areas and drug therapeutic classes driven by similar phenotypes. To that aim, we conducted a network community analysis to detect groups of nodes that are more densely connected with each other than to any other nodes in the network. In total, we identified 65 communities (Figure 2); 24 form the largest component and 41 represent distinct and much smaller components. Afterwards, we annotated disease and drug nodes with anatomical organ system categories using the MeSH and drug ATC classification systems, respectively. Then, we tested the over-representation of these classes within the communities compared to the rest of the network using Fisher's exact test (see Methods). We found that 19 disease classes are enriched in 21 communities and 8 drug classes in 6 communities (Table 2).

**Table 2:** **Enrichment of disease and drug classes in network communities**

**Community**	**Drug ATC Anatomical main group**	**Disease MeSH class**
7	-	Parasitic diseases
16	-	Digestive system diseases
17	*Antineoplastic and immunomodulating agents*	*Immune system diseases*
	Musculo-skeletal system	*Bacterial infections and mycoses*
18	*Sensory organs*	*Eye diseases*
		Virus diseases
25	-	Stomatognathic diseases
28	*Genito urinary system and sex hormones*	*Endocrine system diseases*
	*Systemic hormonal preparations excluding sex hormones*	*Female urogenital diseases and pregnancy complications*
		*Male urogenital diseases*
32	*Nervous system*	*Nervous system diseases*
		*Mental disorders*
		Nutritional and metabolic diseases
37	*Blood and blood forming organs*	*Hemic and lymphatic diseases*
		Female urogenital diseases and pregnancy complications
		Male urogenital diseases
38	-	Respiratory tract infections
39	-	Mental disorders
41	-	Nervous system diseases
51	-	Endocrine system diseases
52	-	Neoplasms
53	-	Nutritional and metabolic diseases
56	-	Nutritional and metabolic diseases
		Digestive system diseases
57	-	Skin and connective tissue diseases
59	-	Endocrine system diseases
		Musculoskeletal diseases
60	-	Stomatognathic diseases
		Musculoskeletal diseases
62	-	Nutritional and metabolic diseases
		Respiratory tract diseases
		Bacterial infections and mycoses
63	-	Otorhinolaryngologic diseases
		Eye diseases
64	*Dermatologicals*	*Skin and connective tissue diseases*

Entries in italics denote drug and disease classes of the same anatomical area enriched in the same community.

### Evaluation of the enrichment of disease and drug classes within communities

Some disease classes, such as 'Nutritional and metabolic diseases' are enriched in several communities. This is likely due to the diversity of drugs causing metabolic effects. Overall, enrichment of drug ATC classes mirrors the enrichment of disease classes within the communities strikingly well. Seven out of 13 ATC classes are co-enriched with 9 clearly related disease classes (Table 2), such as the ATC class 'Nervous system' with the MeSH class 'Nervous system diseases'. This shows that, at least for these drug classes, the side effects tend to be related to the anatomical disease area of drugs' indications.

We also observed that in 11 communities several classes are co-enriched. Related molecular mechanisms underlying diseases and drug targets seem to drive the co-enrichment of disease and drug classes in these communities. For example, in community 28 the co-enrichment of endocrine, urogenital, and pregnancy disorders reflects the role of hormone regulation within these disease classes (Table 2). This is further substantiated by the drug ATC class enrichment of 'sex and non-sex hormones' within this community. In the same line, co-enrichment of endocrine and musculoskeletal disorders agrees with the involvement of the endocrine system in musculoskeletal development [47,48]. Consistent with the adverse metabolic impact of typical and atypical antipsychotics [49,50], nutritional and metabolic disorders are found to be co-enriched with nervous system and mental disorders in community 32. Concerning atypical antipsychotics, this effect has recently been attributed to their antagonistic effect on the muscarinic M3 receptor, which is involved in the regulation of insulin secretion [51]. These examples indicate that related molecular mechanisms drive the associations between drug and disease classes.

### Connections between drug mechanisms of action and disease classes

**Figure 3:**
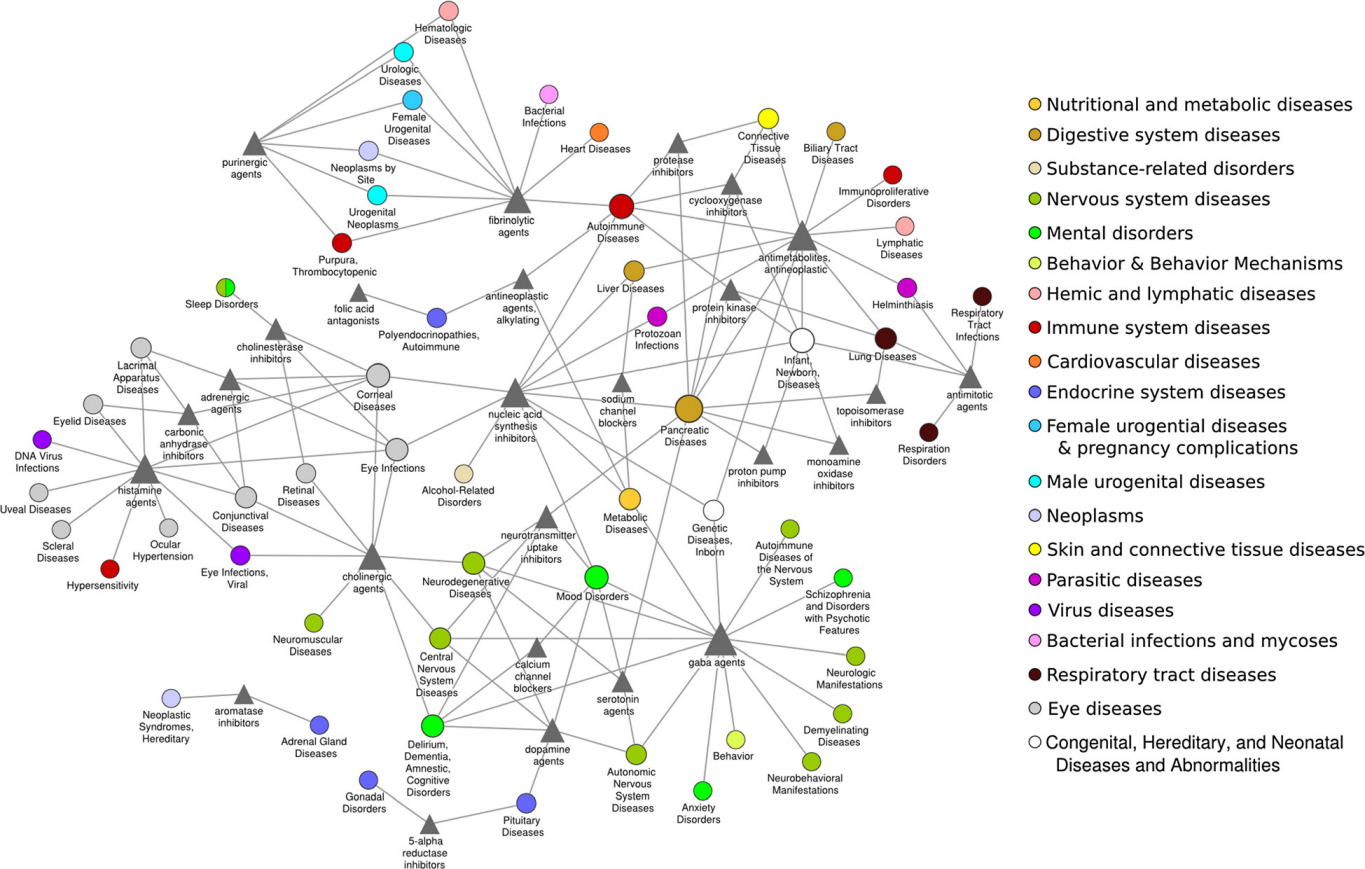
**Phenotypic relationships between therapeutic molecular mechanisms of drugs and disease classes.** Combinations of disease classes (colored circles) and molecular mechanisms of pharmacological action of drugs (triangles) statistically enriched in the phenotypic disease-drug network. The colors of disease nodes represent organ systems and general disorder classes. Node sizes are proportional to the number of links to other classes.

Motivated by our findings that drugs and diseases sharing molecular mechanisms exhibit high phenotypic similarity (Figure 1), we analyzed the phenotypic similarity network with respect to the enrichment of specific combinations of molecular drug mechanisms and disease classes. For this, we utilized Fisher's exact test to determine significantly overrepresented combinations of disease classes and therapeutic mechanisms of action (MoAs) of drugs within the network compared to all possible drug-disease pairs. We found 133 overrepresented combinations that cover 701 (9.5%) disease-drug associations within the phenotypic similarity network. Figure 3 visualizes the results as a network connecting 26 drug MoAs to 55 disease classes.

Below we highlight interesting connections between drug mechanisms of action and disease classes found in this analysis.

### Connections between drug MoAs and multiple disease classes

In this network, several drug MoAs are associated with multiple distinct disorder classes, such as 'nucleic acid synthesis inhibitors', 'antimetabolites, antineoplastic' and 'antimitotic agents', consistent with the impact of these mechanisms on cell cycle regulation. For example, nucleic acid synthesis inhibitors are linked to many different organ-specific disease classes due to the widespread effect of non-specific inhibition of DNA and RNA synthesis. In the same line, antineoplastic antimetabolites are highly connected in the network to diseases affecting cells and tissues with proliferative capacity such as the gastrointestinal tract (biliary system, pancreas, liver), bone marrow, lung, skin and connective tissues, reflecting the adverse impact of these therapeutics on these organ systems [52].

Other drug classes such as histamine and adrenergic agents and 5-alpha reductase and aromatase inhibitors are linked to multiple disorder classes affecting the same organ system, such as numerous eye disorders and various endocrine disorders, respectively. While the localized effects in the eye of histaminergic and adrenergic agents echo the topical application of these drugs on the eyes, the endocrine effects of the latter agents are clearly related to mechanistic drug effects. The association of 5-alpha reductase inhibitors with gonadal disorders mimics the association of antiandrogenic effects of these inhibitors and phenotypes such as eunuchoidism and Reifenstein syndrome. Similarly, the link of 5-alpha reductase inhibitors with pituitary disorders is in line with the observed role of 5-alpha reductase within the feedback control of the hypothalamic-pituitary-gonadal axis [53,54].

### MoAs-disease connections that reflect on-target as well as off-target drug MoAs

The connections of pharmacological agents with disease areas outside their main indication area appear to be related to the expression and activity of their drug targets within these organ systems. For example, in addition to the expected connections of gamma-aminobutyric acid (GABA) agents with nervous system-related disease classes [50,55,56], the recently recognized function of the GABAergic system in autonomic function [57] and metabolic diseases [58] is captured in our network. Likewise, serotonin agents are linked to neurodegenerative diseases affecting the central nervous system (Figure 3) as well as to autonomic nervous system diseases. The latter connection is supported by the presence of serotonin outside of the central nervous system and the regulatory role of the serotonergic system on many autonomic processes such as heart rate, respiration, and gastrointestinal functions [59].

### MoAs-disease connections that reflect side effect of drugs

This network also reinforces the suspected relationship of certain drug MoAs and side effect reactions such as the association between purinergic and fibrinolytic agents with cancer, which has been established only recently [60,61].

### Connections between autoimmune and pancreatic disorders and multiple drug MoAs

Interestingly, autoimmune and pancreatic disorders are disease classes with connections to several drug MoA classes, reflecting the multiplicity of molecular mechanisms affecting these disorders. Autoimmune disorders are linked to different mechanistic classes such as fibrinolytic and antineoplastic agents as well as protease, kinase, and cycloxygenease inhibitors. Curiously, four tyrosine kinase inhibitors and systemic lupus erythematosus (SLE) underline the phenotypic similarity between protein kinase inhibitors and autoimmune diseases in the network. Three of these four drugs inhibit mast cell growth factor receptor tyrosine kinase KIT, suggesting that impaired KIT signaling might play a role in both drug-induced as well as systemic lupus erythematosus.

### Enrichment of clinical disease-drug associations

**Figure 4:**
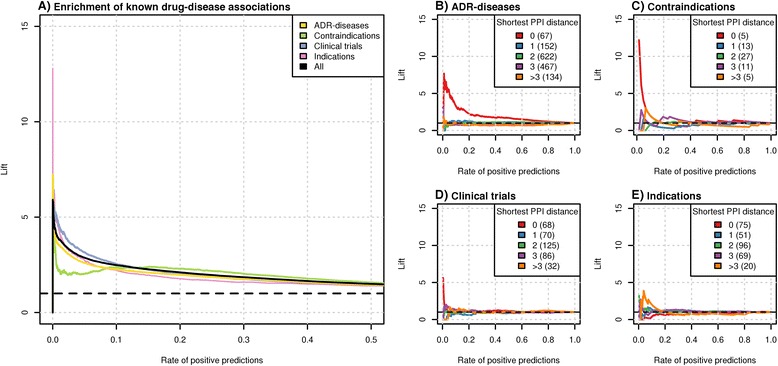
**Phenotypic similarity and clinical disease-drug associations.** A disease-drug pair is considered a known clinical association if treatment of patients affected by this disease with this drug is indicated, contraindicated, has been tested in phase 3 and 4 clinical trials, or represents a case where the disease has been reported as a side effect of the drug (ADR-diseases). **(A)** Enrichment of clinical relationships. 'All' refers to the combined set of the four relation types. Here, each type represents a separate benchmark set. **(B-E)** Enrichment of shortest path lengths among ADR-disease associations **(B)**, contraindications **(C)**, clinical trial associations **(D)** and indications **(E)**. The number of known clinical associations in each distance category is given in parentheses. The dashed black lines reflect performance at random expectation.

The previous observation of co-enrichment of disease classes with drugs indicated to treat disorders affecting the same organ systems raised the question of whether drugs and diseases with similar phenotypes are enriched in known drug-disease associations such as indications and contraindications. To tackle this question, we collected information on several types of drug-disease associations and mapped these to our entire set of disease-drug pairs. This resulted in 2,637 and 2,230 pairs of drugs indicated and contraindicated for diseases, respectively, and 34,467 pairs where the disease has been reported as a side effect of the drug. We termed these associations indications, contraindications and ADR-disease relationships, respectively (see Methods). As a subtype of indication relationships we also collected 7,774 pairs of drugs and diseases tested in clinical trials (phases 3 and 4, when efficacy has already been established). Then, we employed the lift measurement to quantify the enrichment of known drug-disease associations over random expectation for drug-disease pairs sorted by decreasing values of phenotypic similarity. We observed an enrichment of more than 2.5-fold with maximum enrichment of 5.9-fold among the 10% of drug-disease pairs with highest phenotypic similarity score (Figure 4A, all). This shows that clinically related drugs and diseases tend to cause similar phenotypic effects.

Next, we investigated if the different types of known drug-disease relationships differ in the level of enrichment by evaluating each relation type separately. We observed that indications and clinical trial pairs had a slightly stronger tendency to exhibit high phenotypic similarity (lift of 4.3 and 4.6 at 0.01 rate of positive prediction), followed by ADR-disease pairs (3.5-fold) and contraindicated drug-disease (2.2-fold) (see Table S2 in Additional file 2 for details).

### The high phenotypic similarity of contraindications and ADR-disease pairs are likely due to common molecular mechanisms

In order to examine whether common or related molecular mechanisms are associated with phenotypic similarity among clinical relationships, we evaluated the enrichment of related molecular mechanisms individually for 311 indications, 381 clinical trials, 61 contraindications, and 1,442 ADR-disease relationships. Interestingly, for ADR-disease and contraindication pairs we observe a clear enrichment of drugs targeting disease-associated proteins (Figure 4B,C), revealing that the high phenotypic similarity exhibited by contraindications and ADR drug-disease relationships is linked to shared molecular mechanisms. Surprisingly, for phenotypically related indication pairs we observe a weak enrichment only for shortest distances of 3 or higher (Figure 4D,E). The narrow peak for distance 0 for clinical trials represents two exceptions of pairs that share molecular mechanism and phenotypes, namely quetiapine and Parkinson's disease and methylphenidate (ritalin) and bipolar disorder. Overall, we conclude that the similar phenotypes exhibited by drugs and diseases related by indication are unlikely to be due to common molecular mechanisms. This agrees with previous findings that the majority of current drug treatments represent palliative therapeutic interventions aimed at treating disease symptoms rather than their causes [62].

### Drugs with their contraindicated diseases are associated with highest phenotypic similarity

**Figure 5:**
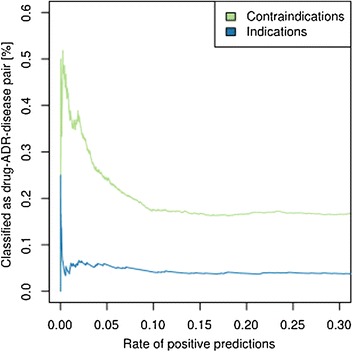
**Fraction of contraindications and indications classified as drug-ADR-disease pairs.** Enrichment among high-scoring disease-drug pairs of the fraction of indications and contraindication also classified as drug-ADR-disease pairs. Each relation type represents a separate benchmark set.

Another interesting observation is that drugs and their contraindicated diseases tend to have higher similarity scores than the other clinical relation types (Benjamini-Hochberg adjusted *P*-values of Wilcoxon tests: 1.5e-21 for contraindication versus indication, 1.5e-02 for contraindication versus clinical trial, and 3.0e-136 for contraindication versus ADR-disease relationships; see also Figure S1 in Additional file 2). This result implies that certain contraindications, that is, those with high semantic similarity score, can be recognized by the great resemblance of the drug's side effects to the symptoms of the contraindicated disease. The drugs in these pairs are thus likely to have a high propensity to cause the disease or to considerably aggravate its symptoms. Indeed, this is confirmed by the rising fraction of ADR-disease relationships observed with increasing semantic similarity values among contraindication relationships. This fraction reaches its highest value (45%) on the top 2% (score ≥1.64) (Figure 5) of contraindications. For example, the contraindication connecting the disease fibrosis and the drug tioguanine is one of these relationships. This disease-drug pair shares specific phenotypic features (Table S3 in Additional file 2) and also represents an ADR-disease connection as the drug is known to cause fibrosis [63]. Interestingly, we found 752 ADR-disease pairs within the top 2% of pairs that have not been recognized as contraindications yet (Additional files 5 and 6). Our results suggest that physicians and drug regulatory agencies should consider these pairs carefully as administration of these drugs to patients suffering from the corresponding disease conditions is potentially inadvisable. In conclusion, we observed that clinically related pairs of diseases and drugs tend to exhibit similar phenotypes, and that drugs with their contraindicated diseases are associated with highest phenotypic similarity. For ADR-disease pairs and contraindications this is likely due to common molecular mechanisms while drugs indicated for diseases tend to be more distantly related on the molecular level.

### Main findings

In summary, the analysis of phenotypic relationships between drugs and diseases have revealed the following scientific findings. First, molecularly or clinically related drugs and diseases tend to exhibit high phenotypic similarity. Second, many drugs and diseases linked to the same organ class are enriched among phenotypically associated disease-drug pairs like the drug class 'Nervous system' and the class 'Nervous system diseases' or 'Sensory organs' and 'Eye diseases'. Third, specific combinations of molecular drug mechanisms and disease classes are phenotypically connected, reflecting, for example, on-target as well as off-target drug MoAs. For example, GABA agents are not only associated with nervous system-related disease classes, but also to autonomic function and metabolic diseases, a function of the GABAergic system that was only recently recognized. Fourth, drugs with their contraindicated diseases are associated with highest phenotypic similarity, which is likely due to common molecular mechanisms.

Taken together, these results contribute to the understanding of drug effects and suggest that phenotypic similarity of drug-disease pairs might be used to propose mechanisms for diseases with yet unknown causes. Moreover, as immediate clinical translation of our analysis, we provide a list of disease-drug pairs where the diseases should be carefully considered as a precaution or potential contraindication for administration of the drugs.

## Discussion

In this work we have used a semantic similarity approach to systematically analyze phenotypic similarity between drugs and diseases. We find that drugs and diseases associated with similar molecular mechanisms, especially those affecting the same protein or interacting proteins, exhibit similar phenotypes. The same observation was made for known drug-disease associations, such as indications, contraindications and drugs that cause the disease as a side effect. The phenotypic similarity of contraindications and ADR-disease relationships clearly originates from shared or very similar perturbed molecular mechanisms. Thus, the assessment of phenotypic similarity of drugs and diseases represents a novel approach to detect mechanistic causes of diseases and side effects.

The analysis of overrepresented combinations of disease classes and molecular mechanisms of drugs shows that the phenotypic impact of certain drug classes is linked either to their therapeutic action (on-targets) or to closely related off-targets. For example, our approach captures the on-target effects of serotonin agents, aromatase and 5-alpha reductase inhibitors and the link between off-target effects of protein kinase inhibitors and SLE. In relation to the latter connection, it has been speculated that unintended modulation of cytoplasmic Src-family tyrosine kinases by certain inhibitors of protein kinases might be responsible for drug-induced lupus erythematosus [64]. In contrast, our analysis suggests that impaired KIT signaling plays a role in both drug-induced as well as SLE. This hypothesis is supported by the significantly lower levels of soluble KIT found in SLE patients compared to controls [65].

This analysis also supports the suspected associations between certain MoAs and diseases, such as the link between fibrinolytic and purinergic agents with cancer. Cancer is connected to several fibrinolytic drugs, including recombinant forms of tissue-type plasminogen activator (t-PA). Consistent with this link, the t-PA serine protease has been shown to promote tumor cell invasion [60] and fibrinolysis has been identified as a risk factor in tumor invasion [66]. Similarly, the purinergic receptors P2Y1 and P2Y2 have been found to exert proliferative as well as antiproliferative effects [67]. These effects are in accordance with the suspected cancer promoter activity of the purinergic drug prasugrel [68]. In agreement with several lines of evidence involving the purinergic adenosine A2a receptor in immune evasion of tumor cells [69–71], we find that regadenoson, an A2a receptor agonist, shares phenotypic features with heart neoplasms. This suggests careful consideration of regadenoson treatment in heart cancer patients.

For other drug classes, the occurrence of certain side effects seems to be linked to the application sites of drug treatments as observed clearly for eye and ophthalmologic agents. The analysis of the phenotypic similarity network reveals that certain diseases and drugs related by indication tend to share phenotypes of disease-associated organs (Table 2). Although we cannot fully explain the resemblance of phenotypic features between drugs and their indicated diseases, possible explanations include the potential bias of reported side effects towards disease symptoms of clinical trial participants or the occurrence of organ- but not disease-specific effects. These organ-specific effects can be captured by the intrinsic property of our semantic similarity approach to measure phenotypic resemblance beyond the detection of identical traits. For example, 'Hypersexuality' is a symptom of bipolar disorder that is semantically similar to the side effect 'Libido decreased' of the drug topiramate that is used to treat this disease. These terms represent two opposing yet related concepts subsumed under 'Sexual desire disorders', thereby contributing to the phenotypic similarity score between bipolar disorder and topiramate. The organ-specific effects might as well reflect a distant but functional relationship between drug targets and disease genes as it is well known that certain diseases such as genetic or chronic disorders like diabetes mellitus require lifelong symptomatic and supportive treatments [72], which do not necessarily target the dysfunctional genes, but can lead to side effects in disease-associated organs.

Although all clinically related drug-disease pairs tend to show high phenotypic similarity, only contraindications and ADR-disease pairs have a propensity to share molecular mechanisms (Figure 4A,B). This clearly suggests a causative connection between molecular perturbation and observed phenotypic similarity in these relationships. Only two pairs investigated in clinical trials show an association between common molecular mechanisms and shared phenotypes. The first pair is quetiapine and Parkinson's disease studied in a phase 4 clinical study [73]. This clinical trial investigated the safety, tolerability, and efficacy of quetiapine in the treatment of psychosis in patients with Parkinson's disease [74,75]. The Parkinson-like effects of quetiapine are likely related to its antagonistic activity on several members of the dopamine receptor family [76–78] as some of these receptors have been associated with Parkinson's disease [79–81]. The second case is formed by the psychostimulant methylphenidate (ritalin) and bipolar disorder. Ritalin is actively studied as a treatment of bipolar disorder or its main symptoms, including mania [82–84]. Ritalin inhibits, amongst other proteins, the same serotonin and dopamine transporters SLC6A4 and SLC6A3 that have been implicated in bipolar disorder [85,86]. In agreement with the phenotypic similarity of this pair, several case reports indicate that ritalin can aggravate bipolar disorder symptoms [87,88]. Besides, ritalin treatment has been associated with younger age of onset of bipolar disorder in predisposed children [89]. This evidence combined with our observation of highly similar phenotypes raise doubts regarding the long-term safety of ritalin in bipolar disorder therapy. The presented examples support the notion that drug-disease pairs with high phenotypic similarity sharing MoAs are likely to correspond to pairs where the administration of the drug to patients diagnosed with the corresponding disease should be avoided.

Another interesting observation concerning contraindications is that these pairs exhibit higher phenotypic similarity than other clinical relationships. This suggests that a drug with high phenotypic similarity to its contraindicated disease is likely to worsen or even to cause the disease condition. We have confirmed this hypothesis by the high proportions of ADR-disease pairs among contraindications with high phenotypic similarity. For example, the ophthalmologic agent medrysone causes uveitis as a side reaction and, consequently, it is contraindicated for this disorder. Another example is the pair formed by haloperidol and Parkinson's disease. Haloperidol, a drug used to treat patients with schizophrenia or related illnesses, might cause parkinsonism [90], a neurological syndrome characterized by Parkinson-like symptoms. Indeed, we observe that 40% of the parkinsonism symptoms, including bradykinesia, cogwheel rigidity, and masked facies, have been reported as side effects of haloperidol. Although many current contraindications might have been proposed based on the recognition of similar clinical phenotypes of drugs and diseases, the systematic comparison of drug and disease phenotypes allows confirmation of this clinical observation and the systematic proposition of many more contraindications.

Unsurprisingly, contraindicated drug-disease pairs with low phenotypic similarity also tend to be highly associated with the risk of causing or worsening the disease as the overall fraction of pairs where the drug is known to cause the disease as a side effect (16.2%) is four times higher than for indications (4.4%). We observed this, for example, for drugs that should not be administered to patients with kidney-related diseases. These contraindications are likely attributable to the kidneys' role in drug metabolism [91] and cannot be anticipated by a semantic similarity approach such as the one presented here.

Curiously, the few indications (113) that are also ADR-disease relationships tend to show high phenotypic similarity as well. Among the top 24 pairs we find 6 beta blockers and 6 other antihypertensive agents linked to heart failure. In 2009, the European Society of Cardiology recommended the use of beta blockers to treat patients undergoing non-cardiac surgery to protect the heart from surgery-related stress. However, there is an ongoing debate whether their antihypertensive effect increases the risk of heart failure [92]. Thus, even for indications, a high drug-disease phenotypic similarity might alert for potential drug-induced aggravation of the disease condition.

## Conclusions

We have systematically analyzed an extensive set of human phenotypes from diseases and drugs and found that molecularly or clinically related drugs and diseases tend to cause similar phenotypes. Specifically, known ADR-disease and contraindication relationships sharing molecular mechanisms exhibit high phenotypic similarity. The analysis of therapeutic molecular mechanisms shows the relationship between certain therapeutic mechanisms and characteristic drug side effects and illustrates that some of these effects are related to the distribution of therapeutic targets across human organ systems. Our results contribute to the understanding of drug effects and suggest that phenotypic similarity of drug-disease pairs might be used to propose mechanisms for diseases with yet unknown causes. Furthermore, identifying disorders that have been reported as potential side effects of a drug and that exhibit high phenotypic similarity to the drug's side effect profile helps to avoid drug treatments that potentially aggravate disease conditions of patients. To facilitate the clinical translation of this finding, we provide a list of disease-drug pairs where the diseases should be carefully considered as a precaution or potential contraindication for administration of the drugs. Overall, the work presented here has important implications in the therapy of diseases as well as in rationalizing drug prescription.

## Additional files

Additional file 1:
**The medical vocabularies included in the UMLS Metathesaurus that were used for the generation of the disease thesaurus.**
Additional file 2:
**Supplementary Figures S1 and S2 and Tables S1, S2 and S3.**
Additional file 3:
**The medical vocabularies included in the UMLS Metathesaurus that were used for the generation of the phenotypic feature thesaurus.**
Additional file 4:
**All SMQs from MedDRA version 13.0 that were used to update the MedDRA ontology with additional 'horizontal' links between the SOC** ***Investigations*** **and the other SOCs.**
Additional file 5:
**Disease-drug pairs with their phenotypic similarity score that should be considered as precautions or potential contraindications.**
Additional file 6:
**Detailed comparison of drug side effects to disease symptoms for drug-disease** **pairs listed in Additional file** **5** **.** For critical assessment of precautions or potential contraindications, we report how drug side effects are related to disease symptoms. 'Phenotypic Commonality (MICA)' reports the most specific term of the MedDRA ontology that relates each disease symptom to the most similar side effect. Highlighted cells indicate shared phenotypic traits. For each disease-drug pair, rows are sorted according to decreasing similarity scores of individual symptom-side effect pairs.

## Abbreviations

ADR: adverse drug reaction
ATC: Anatomical Therapeutic Chemical
ChEBI: Chemical Entities of Biological Interest
CID: compound identifier
EMA: European Medicines Agency
eMC: electronic Medicines Compendium
FDA: US Food and Drug Administration
FDR: false discovery rate
GABA: gamma-aminobutyric acid
IC: information content
LLT: *Low-Level* Term
MedDRA: Medical Dictionary for Regulatory Activities
MeSH: Medical Subject Headings
MICA: most informative common ancestor
MoA: mechanism of action
OMIM: Online Mendelian Inheritance in Man
PPI: protein-protein interaction
PT: *Preferred Term*
SLE: systemic lupus erythematosus
SMQ: Standardized MedDRA Query
SOC: *System Organ Class*

## References

[1] Lamb J, Crawford ED, Peck D, Modell JW, Blat IC, Wrobel MJ, Lerner J, Brunet J-P, Subramanian A, Ross KN, Reich M, Hieronymus H, Wei G, Armstrong SA, Haggarty SJ, Clemons PA, Wei R, Carr SA, Lander ES, Golub TR (2006). The Connectivity Map: using gene-expression signatures to connect small molecules, genes, and disease. Science.

[2] Dudley JT, Deshpande T, Butte AJ (2011). Exploiting drug-disease relationships for computational drug repositioning. Brief Bioinform.

[3] Wang Z-Y, Zhang H-Y (2013). Rational drug repositioning by medical genetics. Nat Biotechnol.

[4] Mestres J, Gregori-Puigjane E, Valverde S, Sole RV (2008). Data completeness: the Achilles heel of drug-target networks. Nat Biotechnol.

[5] Visscher PM, Brown MA, McCarthy MI, Yang J (2012). Five years of GWAS discovery. Am J Hum Genet.

[6] Hidalgo CA, Blumm N, Barabási A-L, Christakis NA (2009). A dynamic network approach for the study of human phenotypes. PLoS Comput Biol.

[7] Blair DR, Lyttle CS, Mortensen JM, Bearden CF, Jensen AB, Khiabanian H, Melamed R, Rabadan R, Bernstam EV, Brunak S, Jensen LJ, Nicolae D, Shah NH, Grossman RL, Cox NJ, White KP, Rzhetsky A (2013). A nondegenerate code of deleterious variants in Mendelian loci contributes to complex disease risk. Cell.

[8] Campillos M, Kuhn M, Gavin A-C, Jensen LJ, Bork P (2008). Drug target identification using side-effect similarity. Science.

[9] Espinosa O, Hancock JM (2011). A gene-phenotype network for the laboratory mouse and its implications for systematic phenotyping. PLoS One.

[10] Van Driel MA, Bruggeman J, Vriend G, Brunner HG, Leunissen JAM (2006). A text-mining analysis of the human phenome. Eur J Hum Genet.

[11] Reyes-Palomares A, Rodríguez-López R, Ranea JAG, Sánchez Jiménez F, Medina MA (2013). Global analysis of the human pathophenotypic similarity gene network merges disease module components. PLoS One.

[12] Ghazvinian A, Noy NF, Musen MA (2009). Creating mappings for ontologies in biomedicine: simple methods work. AMIA Annu Symp Proc.

[13] Washington NL, Haendel MA, Mungall CJ, Ashburner M, Westerfield M, Lewis SE (2009). Linking human diseases to animal models using ontology-based phenotype annotation. PLoS Biol.

[14] Mungall CJ, Gkoutos GV, Smith CL, Haendel MA, Lewis SE, Ashburner M (2010). Integrating phenotype ontologies across multiple species. Genome Biol.

[15] Hoehndorf R, Hiebert T, Hardy NW, Schofield PN, Gkoutos GV, Dumontier M (2014). Mouse model phenotypes provide information about human drug targets. Bioinformatics.

[16] Kuhn M, Szklarczyk D, Franceschini A, Campillos M, von Mering C, Jensen LJ, Beyer A, Bork P (2010). STITCH 2: an interaction network database for small molecules and proteins. Nucleic Acids Res.

[17] Bolton EE, Wang Y, Thiessen PA, Bryant SH (2008). Chapter 12 PubChem: Integrated Platform of Small Molecules and Biological Activities. Annu Rep Comput Chem.

[18] Wishart DS, Knox C, Guo AC, Cheng D, Shrivastava S, Tzur D, Gautam B, Hassanali M (2008). DrugBank: a knowledgebase for drugs, drug actions and drug targets. Nucleic Acids Res.

[19] Kanehisa M, Goto S (2000). Kyoto Encyclopedia of Genes and Genomes. Nucleic Acids Res.

[20] **FDA Substance Registration System.** [http://fdasis.nlm.nih.gov/srs/srs.jsp]

[21] **electronic Medicines Compendium (eMC).** [http://www.medicines.org.uk/emc/]

[22] Lang F, Aronson A: **Filtering the UMLS® Metathesaurus® for MetaMap, 2010 Edition.**ᅟ[http://skr.nlm.nih.gov/papers/references/filtering10.pdf]

[23] Xu R, Musen MA, Shah NH (2010). A comprehensive analysis of five million UMLS Metathesaurus terms using eighteen million MEDLINE citations. AMIA Annu Symp Proc.

[24] Hettne KM, van Mulligen EM, Schuemie MJ, Schijvenaars BJ, Kors JA (2010). Rewriting and suppressing UMLS terms for improved biomedical term identification. J Biomed Semantics.

[25] **The Merck Manual: The Merck Manual of Diagnosis and Therapy**. [http://www.merckmanuals.com/professional]

[26] **The Merck Manual Home Health Handbook**. [http://www.merckmanuals.com/home]

[27] Medical Encyclopedia: **MedlinePlus.** [http://www.nlm.nih.gov/medlineplus/encyclopedia.html]

[28] CureResearch.com: **Symptoms, Diseases and Diagnosis.** [http://www.cureresearch.com/]

[29] Szklarczyk D, Franceschini A, Kuhn M, Simonovic M, Roth A, Minguez P, Doerks T, Stark M, Muller J, Bork P, Jensen LJ, von Mering C (2011). The STRING database in 2011: functional interaction networks of proteins, globally integrated and scored. Nucleic Acids Res.

[30] Von Mering C, Jensen LJ, Snel B, Hooper SD, Krupp M, Foglierini M, Jouffre N, Huynen MA, Bork P (2005). STRING: known and predicted protein-protein associations, integrated and transferred across organisms. Nucleic Acids Res.

[31] Brouwers L, Iskar M, Zeller G, van Noort V, Bork P (2011). Network neighbors of drug targets contribute to drug side-effect similarity. PLoS One.

[32] Bauer-Mehren A, Rautschka M, Sanz F, Furlong LI (2010). DisGeNET: a Cytoscape plugin to visualize, integrate, search and analyze gene-disease networks. Bioinformatics.

[33] Bauer-Mehren A, Bundschus M, Rautschka M, Mayer MA, Sanz F, Furlong LI (2011). Gene-disease network analysis reveals functional modules in mendelian, complex and environmental diseases. PLoS One.

[34] Kuhn M, Szklarczyk D, Franceschini A, von Mering C, Jensen LJ, Bork P (2012). STITCH 3: zooming in on protein-chemical interactions. Nucleic Acids Res.

[35] Clinical Trials Transformation Initiative: **Database for Aggregate Analysis of ClinicalTrials.gov.** [http://www.ctti-clinicaltrials.org/what-we-do/analysis-dissemination/state-clinical-trials/aact-database]

[36] Resnik P (1999). Semantic similarity in a taxonomy: an information-based measure and its application to problems of ambiguity in natural language. J Artif Intell Res.

[37] Pesquita C, Faria D, Falcão AO, Lord P, Couto FM (2009). Semantic similarity in biomedical ontologies. PLoS Comput Biol.

[38] Seco N, Veale T, Hayes J, Lopez de Mántaras R, Saitta L (2004). An intrinsic information content metric for semantic similarity in WordNet. ECAI2004, Proceedings of the 16th European Conference on Artificial Intelligence.

[39] Berthold MR, Cebron N, Dill F, Gabriel TR, Kötter T, Meinl T, Ohl P, Thiel K, Wiswedel B (2009). KNIME - the Konstanz information miner. ACM SIGKDD Explor Newsl.

[40] Csardi G, Nepusz T: **The igraph software package for complex network research.** *InterJournal Complex Syst* 2006 :1695.

[41] Blondel VD, Guillaume J, Lambiotte R, Lefebvre E (2008). Fast unfolding of communities in large networks. J Stat Mech Theory Exp.

[42] Newman MEJ (2006). Modularity and community structure in networks. Proc Natl Acad Sci U S A.

[43] Girvan M, Newman MEJ (2002). Community structure in social and biological networks. Proc Natl Acad Sci U S A.

[44] WHO Collaborating Centre for Drug Statistics Methodology: **ATC/DDD Index.** [http://www.whocc.no/atc_ddd_index/]

[45] Resnik P, Mellish CS (1995). Using information content to evaluate semantic similarity in a taxonomy. Proceedings of the 14th International Joint Conference on Artificial Intelligence. Volume 1.

[46] Pavan M, Todeschini R: *Scientific Data Ranking Methods - Theory and Applications. Volume 27.* Elsevier; 2008:51–72 [Data Handling in Science and Technology].

[47] Ruff RL, Weissmann J (1988). Endocrine myopathies. Neurol Clin.

[48] Waung JA, Bassett JHD, Williams GR (2012). Thyroid hormone metabolism in skeletal development and adult bone maintenance. Trends Endocrinol Metab.

[49] Savoy YE, Ashton MA, Miller MW, Nedza FM, Spracklin DK, Hawthorn MH, Rollema H, Matos FF, Hajos-Korcsok E (2010). Differential effects of various typical and atypical antipsychotics on plasma glucose and insulin levels in the mouse: evidence for the involvement of sympathetic regulation. Schizophr Bull.

[50] Boyda HN, Procyshyn RM, Pang CCY, Hawkes E, Wong D, Jin CH, Honer WG, Barr AM (2013). Metabolic side-effects of the novel second-generation antipsychotic drugs asenapine and iloperidone: a comparison with olanzapine. PLoS One.

[51] Weston-Green K, Huang X-F, Deng C (2013). Second generation antipsychotic-induced type 2 diabetes: a role for the muscarinic M3 receptor. CNS Drugs.

[52] Kumar V, Abbas AK, Aster JC (2012). Robbins Basic Pathology: With STUDENT CONSULT Online Access.

[53] Nagamoto A, Noguchi K, Murai T, Kinoshita Y (1994). Significant role of 5α‐reductase on feedback effects of androgen in rat anterior pituitary cells demonstrated with a nonsteroidal 5α‐reductase inhibitor ONO‐3805. J Androl.

[54] Klein CE, Kufe DW, Pollock RE, Weichselbaum RR, Bast RC, Gansler TS, Holland JF, Frei E (2003). Gonadal Complications. Holland-Frei Cancer Medicine.

[55] Markou A, Cryan J, Möhler H (2012). The GABA system in anxiety and depression and its therapeutic potential. Neuropharmacology.

[56] Kumar K, Sharma S, Kumar P, Deshmukh R (2013). Therapeutic potential of GABAB receptor ligands in drug addiction, anxiety, depression and other CNS disorders. Pharmacol Biochem Behav.

[57] Gladkevich A, Korf J, Hakobyan VP, Melkonyan KV (2006). The peripheral GABAergic system as a target in endocrine disorders. Auton Neurosci.

[58] Hyland NP, Cryan JF (2010). A gut feeling about GABA: focus on GABA(B) receptors. Front Pharmacol.

[59] Berger M, Gray JA, Roth BL (2009). The expanded biology of serotonin. Annu Rev Med.

[60] Díaz VM, Hurtado M, Thomson TM, Reventós J, Paciucci R (2004). Specific interaction of tissue-type plasminogen activator (t-PA) with annexin II on the membrane of pancreatic cancer cells activates plasminogen and promotes invasion in vitro. Gut.

[61] Di Virgilio F (2012). Purines, purinergic receptors, and cancer. Cancer Res.

[62] Yıldırım MA, Goh K-I, Cusick ME, Barabási A-L, Vidal M (2007). Drug-target network. Nat Biotechnol.

[63] Geller SA, Dubinsky MC, Poordad FF, Vasiliauskas EA, Cohen AH, Abreu MT, Tran T, Martin P, Vierling JM, Targan SR (2004). Early hepatic nodular hyperplasia and submicroscopic fibrosis associated with 6-thioguanine therapy in inflammatory bowel disease. Am J Surg Pathol.

[64] Rea D, Bergeron A, Fieschi C, Bengoufa D, Oksenhendler E, Dombret H (2008). Dasatinib-induced lupus. Lancet.

[65] Kitoh T, Ishikawa H, Sawada S, Koshino K, Tokano Y, Hashimoto H, Nakagawa S (1998). Significance of stem cell factor and soluble KIT in patients with systemic lupus erythematosus. Clin Rheumatol.

[66] Zorio E, Gilabert-Estellés J, España F, Ramón LA, Cosín R, Estellés A (2008). Fibrinolysis: the key to new pathogenetic mechanisms. Curr Med Chem.

[67] Burnstock G (2006). Pathophysiology and therapeutic potential of purinergic signaling. Pharmacol Rev.

[68] Floyd JS, Serebruany VL (2010). Prasugrel as a potential cancer promoter: review of the unpublished data. Arch Intern Med.

[69] Ohta A, Gorelik E, Prasad SJ, Ronchese F, Lukashev D, Wong MKK, Huang X, Caldwell S, Liu K, Smith P, Chen J-F, Jackson EK, Apasov S, Abrams S, Sitkovsky M (2006). A2A adenosine receptor protects tumors from antitumor T cells. Proc Natl Acad Sci U S A.

[70] Hoskin DW, Mader JS, Furlong SJ, Conrad DM, Blay J (2008). Inhibition of T cell and natural killer cell function by adenosine and its contribution to immune evasion by tumor cells (Review). Int J Oncol.

[71] Fishman P, Bar-Yehuda S, Synowitz M, Powell JD, Klotz KN, Gessi S, Borea PA: **Adenosine receptors and cancer.** *Handb Exp Pharmacol* 2009 :399–441. 10.1007/978-3-540-89615-9_14 PMC3598010 19639290

[72] el-Hazmi MA (1999). Spectrum of genetic disorders and the impact on health care delivery: an introduction. East Mediterr Health J.

[73] ClinicalTrials.gov: **Treatment of Agitation/Psychosis in Dementia/Parkinsonism (TAP/DAP).** [http://clinicaltrials.gov/ct2/show/NCT00043849]

[74] Shotbolt P, Samuel M, David A (2010). Quetiapine in the treatment of psychosis in Parkinson’s disease. Ther Adv Neurol Disord.

[75] Goldman JG, Holden S (2014). Treatment of psychosis and dementia in Parkinson’s disease. Curr Treat Options Neurol.

[76] Sommer BR (2001). Quetiapine-induced extrapyramidal side effects in patients with Parkinson’s disease: case report. J Geriatr Psychiatry Neurol.

[77] Rummel-Kluge C, Komossa K, Schwarz S, Hunger H, Schmid F, Kissling W, Davis JM, Leucht S (2012). Second-generation antipsychotic drugs and extrapyramidal side effects: a systematic review and meta-analysis of head-to-head comparisons. Schizophr Bull.

[78] Kooij JS, Boonstra AM, Vermeulen SH, Heister AG, Burger H, Buitelaar JK, Franke B (2008). Response to methylphenidate in adults with ADHD is associated with a polymorphism in SLC6A3 (DAT1). Am J Med Genet B Neuropsychiatr Genet.

[79] Ko JH, Antonelli F, Monchi O, Ray N, Rusjan P, Houle S, Lang AE, Christopher L, Strafella AP (2013). Prefrontal dopaminergic receptor abnormalities and executive functions in Parkinson’s disease. Hum Brain Mapp.

[80] Worth PF (2013). How to treat Parkinson’s disease in 2013. Clin Med.

[81] Nolan YM, Sullivan AM, Toulouse A (2013). Parkinson’s disease in the nuclear age of neuroinflammation. Trends Mol Med.

[82] Carlson PJ, Merlock MC, Suppes T (2004). Adjunctive stimulant use in patients with bipolar disorder: treatment of residual depression and sedation. Bipolar Disord.

[83] ClinicalTrials.gov: **Concerta in the Treatment of ADHD in Youth and Adults With Bipolar Disorder.** [http://clinicaltrials.gov/ct2/show/NCT00181987]

[84] ClinicalTrials.gov: **Methylphenidate for the Treatment of Acute Mania.** [http://clinicaltrials.gov/ct2/show/NCT01541605]

[85] Mick E, Kim JW, Biederman J, Wozniak J, Wilens T, Spencer T, Smoller JW, Faraone SV (2008). Family based association study of pediatric bipolar disorder and the dopamine transporter gene (SLC6A3). Am J Med Genet B Neuropsychiatr Genet.

[86] Serretti A, Mandelli L (2008). The genetics of bipolar disorder: genome 'hot regions', genes, new potential candidates and future directions. Mol Psychiatry.

[87] Koehler-Troy C, Strober M, Malenbaum R (1986). Methylphenidate-induced mania in a prepubertal child. J Clin Psychiatry.

[88] Rosse RB, Johri SK, Deutsch SI (1997). Pupillary changes associated with the development of stimulant-induced mania: a case report. Clin Neuropharmacol.

[89] DelBello MP, Soutullo CA, Hendricks W, Niemeier RT, McElroy SL, Strakowski SM (2001). Prior stimulant treatment in adolescents with bipolar disorder: association with age at onset. Bipolar Disord.

[90] Susatia F, Fernandez HH (2009). Drug-induced parkinsonism. Curr Treat Options Neurol.

[91] Lohr JW, Willsky GR, Acara MA (1998). Renal drug metabolism. Pharmacol Rev.

[92] Vogel G (2014). Suspect drug research blamed for massive death toll. Science.

