# Supplementary material for: Molecularly and clinically related drugs and diseases are enriched in phenotypically similar drug-disease pairs
Source: Genome Med. 2014 Aug 17;6(7):52. doi: 10.1186/s13073-014-0052-z (PMC4165361; doi:10.1186/s13073-014-0052-z)
Supplement: Additional file 2: — Supplementary Figures S1 and S2 and Tables S1, S2 and S3. [file 13073_2014_52_MOESM2_ESM.doc]

Supplementary Information

Molecularly and clinically related drugs and diseases are enriched in phenotypically similar drug-disease pairs

**Authors:** Ingo Vogt^1,2^, Jeanette Prinz^1,2^, Monica Campillos^1,2^*

**Affiliations:**

^1^Institute of Bioinformatics and Systems Biology, Helmholtz Zentrum München, 85764 Neuherberg, Germany.

^2^German Center for Diabetes Research, Helmholtz Zentrum München, 85764 Neuherberg, Germany.

***To whom correspondence should be addressed:** [**monica.campillos@helmholtz-muenchen.de**](mailto:monica.campillos@helmholtz-muenchen.de)

**
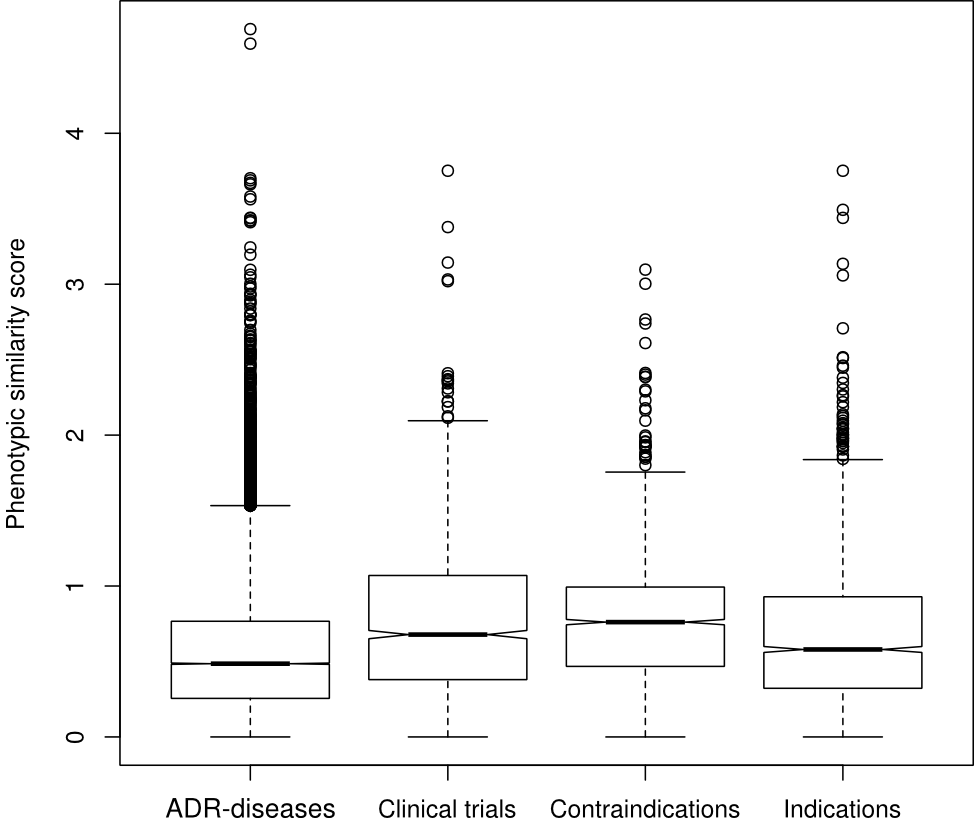
**

**Fig. S1.** Distribution of phenotypic similarity scores for individual clinical association types.


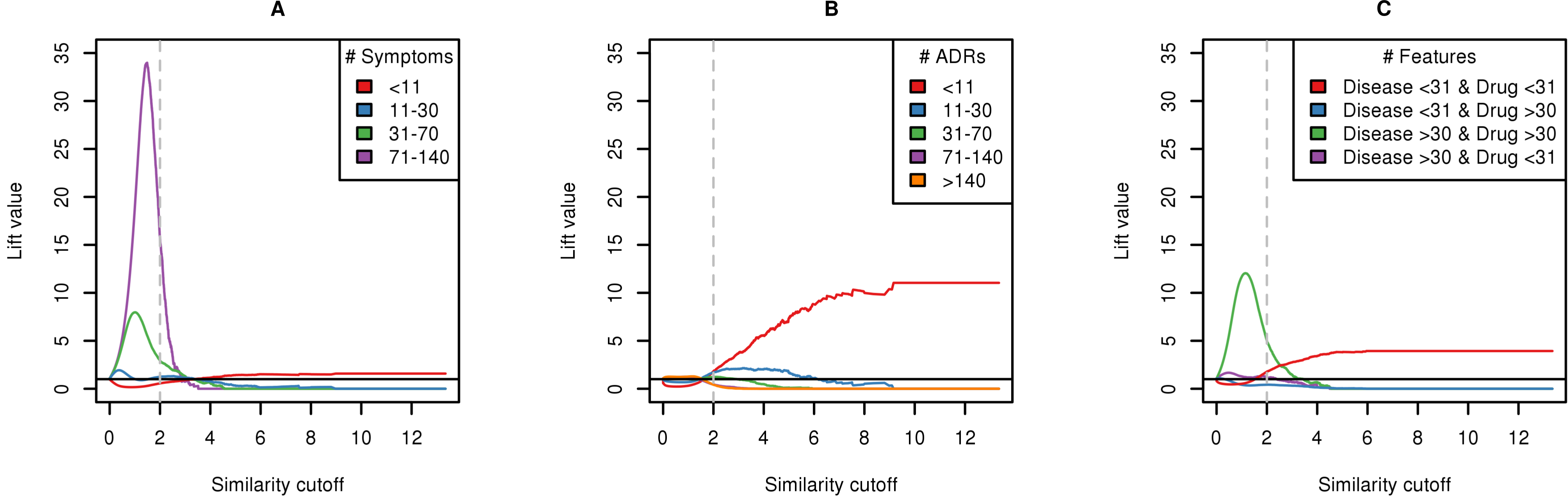
**Fig. S2.** Enrichment of diseases, drugs, and drug-disease pairs for increasing phenotypic similarity. **(A)** Diseases are grouped according to the number of their symptoms. Diseases with more than 30 symptoms are enriched among drug-disease pairs with similarity scores between 1 and 2. From a score of 3 onward only diseases with few symptoms are slightly enriched. **(B)** Drugs were grouped based on the number of their side effects similar to diseases. Unlike diseases, drugs show no enrichment in drug-disease pairs with similarity score up to 2. Above a score of 2, drugs with few ADRs show a clear enrichment. **(C)** Based on the former plots, drugs and diseases were grouped in two categories each, and the enrichment of the combinations of these categories was plotted. When both drug and disease have rather higher numbers of features, they are more likely to obtain higher scores, up to a score of approx. 3. Starting from a score of 2, drug-disease pairs with few numbers of features on both sides are enriched. The horizontal black line indicates the enrichment of 1 expected at random. The vertical dashed gray line at x=2 indicates the similarity score cut-off used for the generation of the phenotypic similarity network.

| **Chemicals name sources** | **Added via** | **# Contributed names** | **Proportion [%]** |
| --- | --- | --- | --- |
| STITCH | CID | 122,637 | 77.65 |
| UNII | Preferred name | 26,434 (776) | 16.74 |
| UNII | Synonym | 4,234 (4,035) | 2.68 |
| KEGG | KEGG ID | 1,598 | 1.01 |
| DrugBank | CID | 1,360 | 0.86 |
| DrugBank | Synonym | 215 (212) | 0.14 |
| KEGG | Preferred name | 1,109 (8) | 0.70 |
| Manual | *Manually* | 159 (82) | 0.10 |
| KEGG | Synonym | 74 (68) | 0.05 |
| ATC | Preferred name | 49 (47) | 0.03 |
| DrugBank | Preferred name | 40 | 0.03 |
| DrugBank | KEGG ID | 15 | 0.01 |
| DrugBank | ChEBI ID | 4 | 0.00 |
| Pubchem | CID | 2 | 0.00 |

**Table S1.** Sources of chemicals names in our drug thesaurus. These names were used to identify drugs described in electronic documents such as drug labels. STITCH was used as base thesaurus which was then extended with data from additional name sources. Values given in parentheses state the number of names assigned to one of the newly created 832 drug thesaurus records not linked to STITCH data.

| Association type | Lift at 0.1 Rpp | Lift at 0.01 Rpp | Maximum lift |
| --- | --- | --- | --- |
| All | 2.48 | 3.83 | 5.91 |
| Indications | 2.21 | 4.29 | 12.80 |
| Clinical trials | 2.54 | 4.55 | 7.23 |
| Contraindications | 2.37 | 2.20 | 7.03 |
| ADR-diseases | 2.26 | 3.53 | 7.24 |

**Table S2.** Enrichment of clinical associations with increasing phenotypic similarity. Enrichment was measured as lift at 0.1 and 0.01 of rate of positive prediction (Rpp), referring to the top 10% and 1% of highest scoring drug-disease pairs.

| Fibrosis symptoms | Tioguanine side effects | Most specific relationship |
| --- | --- | --- |
| Portal hypertension | Portal hypertension | *exact match* |
| Splenomegaly | Splenomegaly | *exact match* |
| Ascites | Ascites | *exact match* |
| Hepatic cirrhosis | Nodular regenerative hyperplasia | Hepatic fibrosis and cirrhosis |
| Hepatic fibrosis | Nodular regenerative hyperplasia | Hepatic fibrosis and cirrhosis |
| Hepatic encephalopathy | Ascites | Hepatic and hepatobiliary disorders |
| Gastrointestinal haemorrhage | Gastrointestinal necrosis | Vascular disorders |

**Table S3.** Symptoms of fibrosis and the most similar side effects of tioguanine.
